# Counting fish at night using artificial light: transect survey of common bream *Abramis brama* and northern pike *Esox lucius* abundance as an example

**DOI:** 10.1111/jfb.15988

**Published:** 2024-11-12

**Authors:** Aatu Turunen, Niko Lappalainen, Hannu Huuskonen, Anssi Vainikka

**Affiliations:** ^1^ Department of Environmental and Biological Sciences University of Eastern Finland Joensuu Finland; ^2^ School of Computing University of Eastern Finland Joensuu Finland

**Keywords:** abundance, autumn, littoral zone, water temperature

## Abstract

Visual transect counting of large‐bodied fish using artificial light at night in a shallow littoral zone (<1 m water depth) is introduced as a complementary survey method for fishes such as common bream *Abramis brama* and northern pike *Esox lucius* that are under‐represented in standard gillnet surveys. The results suggest that transect counting at night and applying necessary corrections for environmental variables could provide a simple and repeatable method to assess the presence and abundance of large‐bodied fishes in lakes with satisfactory water clarity.

Standardized multipanel survey gillnets such as Nordic multimesh gillnets are commonly used to obtain information about fish community composition (Appelberg et al., 1995; Jurvelius et al., 2011; Olin et al., 2016). However, the survey gillnets are ineffective to estimate the abundance of large‐bodied fishes such as common bream *Abramis brama* L. and northern pike *Esox lucius* L. due to poor catchability and the relative rarity of large individuals (Hamley, 1975; Olin et al., 2009; Olsson, 2019). Alternative methods such as trawling, hydroacoustics, and electrofishing (Jurvelius et al., 2005; Rask et al., 2020; Sutela et al., 2008) are costly, time‐consuming, and often also ineffective for assessing species and size classes that inhabit littoral areas. As such, development of new and cheap, low labour‐intensive methods that would be accessible even to citizen scientists could provide practical tools for monitoring the presence and abundance of large‐bodied lake fishes (Olin et al., 2009; Olsson, 2019). Direct visual counting methods have been developed and used for species such as Eurasian perch *Perca fluviatilis* L. (Viljanen & Holopainen, 1982), Atlantic salmon *Salmo salar* L. (Locke, 1997), and *E. lucius* (Turner & Mackay, 1985). These methods are utilized to derive population density metrics but also to gain insight into the habitat choice and ecology of large‐bodied species (Locke, 1997; Turner & Mackay, 1985). Since the direct counting is dependent on water being transparent enough to observe the fish before their fleeing, the applications of direct counting methods are limited to clear‐water lakes and streams (Locke, 1997; Turner & Mackay, 1985).

*Abramis brama* and *E. lucius* are widely occurring species and common also in the Nordic region (Lehtonen et al., 2008; Spens & Ball, 2008). *Abramis brama* is an ecologically important part of lake and river ecosystems, as it moves and suspends sediments during feeding (Lammens et al., 2002; Smith, 2019) whereas *E. lucius* is the typical apex predator in boreal lakes (Spens & Ball, 2008). Although both species are ecologically important, the knowledge of especially *A. brama* populations in lakes is sparse and dependent on studies utilizing large trawls, fyke traps, or electrofishing (Rask et al., 2020; Říha et al., 2015). *A. brama* is suggested to inhabit deeper water layers during daytime and move closer to the shore at night (Muška et al., 2013; Říha et al., 2015; Schulz & Berg, 1987), whereas *E. lucius* efficiently utilizes both pelagic and littoral habitats (Chapman & Mackay, 1984; Říha et al., 2015). The tendency of large‐bodied fish to enter shallow littoral areas in late autumn has been utilized also in traditional Finnish night‐time trident fishing (e.g., *tuulastus* or *tuohestus* in Finnish), in which the immobile fish are killed by directly spearing them under artificial light. Such behavioral tendencies of *A. brama* (Schulz & Berg, 1987) and *E. lucius* (Diana, 1980) could be utilized also for population assessment purposes. Here, a night‐time visual transect counting method is introduced for fishes that use shallow littoral habitats in late autumn during the dark period. We applied the method for *A. brama* and *E. lucius*, and examined if the observable counts of fish are repeatable and affected by environmental variables such as water temperature, wind speed, and vegetation coverage.

Counting trials were performed on seven individual occasions between September and October 2023 in shallow littoral sections, assessed suitable for testing the method, of the large clear‐water (Secchi depth = 2.5 m, measured from 1.5 km distance to the transects in 2018 [Finnish Environmental Institute, 2023]) Lake Höytiäinen in Eastern Finland (62.82°N, 29.65°E) (Huuskonen et al., 2019). Counting transects were allocated to eight sites to cover a total of 2.3 km of the shoreline. The length of transects was 300 m (*n* = 5), 250 m (*n* = 1), or 200 m (*n* = 2) to avoid disturbance to shoreline property owners. The predetermined transects (Figure 1) were examined using a rowing boat and an LED light (HELLA 1GA 357,106,022, 25 W, 2500 lm) connected to a 12‐volt lead‐acid battery. During the counting, the maximum distance between the rowing boat and the shoreline was ca. 10 m and the maximum water depth was 1 m. When counting, a scorekeeper stood on the front bench of the rowing boat and observed the environment at the front of the boat for the 3‐m wide counting area as determined by the cone of the LED light. Counting was initiated between 1 and 2 h after sunset to ensure the necessary darkness. Counting was performed in shifts by two people by altering roles at each new transect. The average time taken to count a transect was 13 ± 3 min (average ± standard deviation [SD]) and the speed was 1.29 ± 0.26 km h^−1^. The density of fish per hectare was calculated by dividing the number of observed fish by the observed area. A sample of five *A. brama* was fished using an eight‐spiked trident to confirm species identification and measure the size of the typically observed fish (see the Ethical statement). During the counting period, the water temperature decreased from 17.3 to 2.4°C until ice cover prevented observations on October 23, 2023.

**FIGURE 1:**
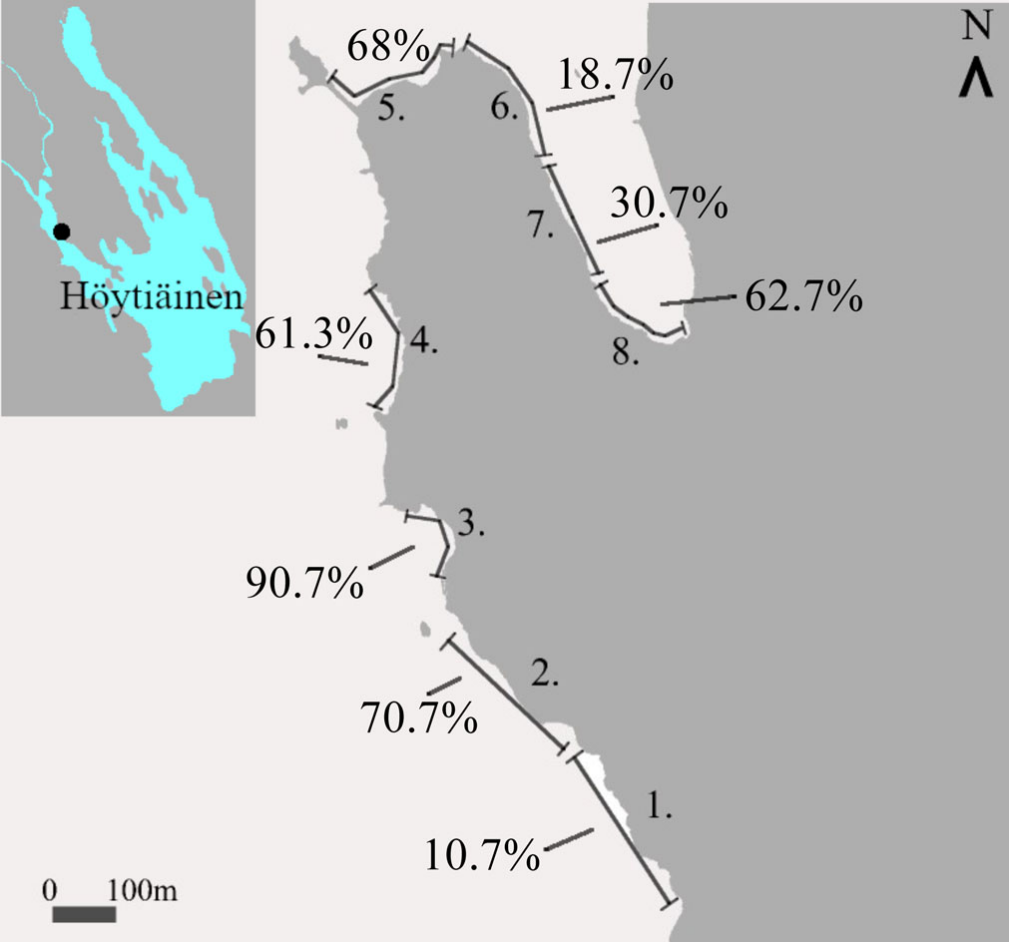
Map of the study transects in Lake Höytiäinen with the estimated vegetation cover in the transects. The presence of pike and break was estimated in these transects by visual night‐time counting using artificial light. The map was modified from a topographic map obtained from National Land Survey of Finland, 10/2023 topographic map set (CC 4.0) (National Land Survey of Finland, 2023).

Water temperature was measured in the middle of each transect during counting trials using a digital thermometer with 0.1°C resolution. Measurements were taken 0.15 m below the surface. Wind speed (m s^−1^) data were collected from the Finnish Meteorological Institute's database (https://en.ilmatieteenlaitos.fi/download-observations) representing a close location (22 km) at Liperi, Joensuu airport (62.66°N, 29.61°E) at an hourly resolution (Finnish Meteorological Institute, 2023, accessed 1.11.2023). Counting trials were pre‐scheduled so that the forecast for the wind speed was not over 3 m s^−1^ to limit water surface movement that could affect visibility and the ability to observe fish.

The relative coverage of aquatic vegetation was estimated on September 28, 2023 (the fourth counting occasion) from three photographs of 1 m^2^ squares in every counting transect. The location of individual squares was selected using a timer and alarm to haphazardly cast a 1‐m measuring plank to the left side of the rowing boat in 3‐min intervals during the fish counting session. The distance of the first photograph from the starting point was approximately the same as that of the last photograph to the end point. The photographs of the measuring plank in the water were later used to form 1 m^2^ squares that were further divided into grids of 0.2 × 0.2 m, and the number of grid squares (three photographs × 25 squares per photograph = 75 squares total) with pieces of aquatic plants was counted.

Accurate lengths for each counting transect were measured using an open‐source wristwatch Bangle.js 2 (Bangle, 2023) (MIT Licence) with location tracking frequency set to 10 s. Data exported from the watch displayed latitude and longitude with six decimal accuracy (accuracy of 0.1 m). Observed distances were used as offsets in fitting linear mixed‐effect (LME) models and forming fish per hectare estimates.

The relationship between environmental variables and fish abundance was studied using generalized LME models with a negative binomial error distribution. In the model, the intercept for each transect was modelled as a random factor, and the fixed variables were water temperature (°C), vegetation cover (proportion), wind speed (m s^−1^), and a binary water clarity factor (vision to bottom/no vision to bottom). Binary water transparency was used to account for stormy preceding days making the water turbid. Accurate counting transect lengths were used as offsets in the models. The best models were selected by backward selection, reducing the least significant variables by the *p* value (*α* = 0.05). The models were fitted using the *glmmTMB* package (Brooks et al., 2017) in R (4.3.2). The variance explained (pseudo *R* ^2^) by the entire model (conditional *R* ^2^) and the model solely with the fixed variables (marginal *R* ^2^) were assessed using the *MuMin* package (Bartoń, 2023) in R.

The repeatability of the fish abundance was estimated using the intraclass correlation coefficient (ICC, Lessells & Boag, 1987) calculated using the *irr* package (Gamer et al., 2019) (since trials with Poisson‐distributed data and inclusion of transects as random factors did not converge in *rptR*) in R for the full dataset and for the dataset without the last two problematic occasions (October13 and 18) removed. The ICC values were calculated using a two‐way model and single‐rater unit (Shrout & Fleiss, 1979). ICC describes the repeatability of observed density by comparing the between‐transect density with the total variation in all density.

To compare the performance of the night counting in relation to standard multimesh gillnet surveys, local Nordic gillnet data (51 gillnet nights in total, six gillnets within a 1.5‐km radius from the present study area) obtained in 2015 (Paajanen & Kiiskinen, 2015) and 2024 (Huuskonen unpublished) and more distant data (radius = 12.5 km) (Paajanen & Ahosola, 2023) were used. In 2015, total of seven *A. brama* were observed with the number per unit effort (NPUE) = 0.13 ind. gillnet^−1^ night^−1^, but *E. lucius* was absent (Paajanen & Kiiskinen, 2015). Within a 1.5‐km radius of our counting transects, two small *A. brama* were observed (fresh mass 45 and 40 g) in 2015 and five bream (average fresh mass 100 g) and zero pike in 2024. In the 2023 data, total of 102 *A. brama* were caught with NPUE = 2.0 ind. gillnet^−1^ night^−1^ and three *E. lucius* with NPUE = 0.1 ind. gillnet^−1^ night^−1^ (Paajanen & Ahosola, 2023).

A total of 94 *A. brama* and 45 *E. lucius* were observed in the study. The average density of *A. brama* was 19.6 ± 23.8 (average ± SD) ind. ha^−1^, while the average density of *E. lucius* was 9.4 ± 14.9 (average ± SD) ind. ha^−1^. The average length of captured *A. brama* (*n* = 5) was 317 ± 49 (average ± SD) mm, and fresh mass was 336 ± 177 (average ± SD) g. *E. lucius* body lengths were visually approximated to range from 200 to 700 mm.

The best explanatory model based on AIC (Akaike information criterion) for *A. brama* abundance consisted of a binary water clarity variable (*β* = −1.79, Standard error (S.E.) = 0.46, *Z* = −3.87, *p* < 0.001; Table 1). The best explanatory model for *E. lucius* abundance comprised wind speed and water temperature (Table 1). Wind speed had a negative effect on *E. lucius* abundance (*β* = −0.29, SE = 0.04, *Z* = −2.06, *p* = 0.040) while water temperature had a positive effect (*β* = 0.12, SE = 0.05, *Z* = 2.56, *p* = 0.010). Vegetation coverage had no effect on the abundance of either species.

**TABLE 1:** Negative binomial mixed models explaining the abundance *A. brama* and *E. lucius* in the studied transects in relation to environmental variables.

*Abramis brama*: t1 (transect 1): 7, 0, 0, 2, 1, 0, 0; t2: 0, 2, 1, 0, 2, 0, 0; t3: 2, 1, 2, 0, 4, 0, 0; t4: 0, 0, 0, 0, 0, 0, 1; t5: 9, 3, 3, 0, 4, 0, 0; t6: 5, 3, 2, 2, 3, 0, 0; t7: 2, 6, 2, 0, 0, 1, 0; t8: 4, 4, 5, 1, 6, 3, 1					
Model	AIC	Reduced	*p* value	*R* ^2^ marginal	*R* ^2^ conditional
~Vegetation + temperature + wind speed + clarity + (1|transect)	185.9	Vegetation	0.982	0.35	0.46
~Temperature + wind speed + clarity + (1|transect)	183.9	Temperature	0.740	0.35	0.45
~Wind speed + clarity + (1|transect)	182.0	Wind speed	0.137	0.35	0.45
**~Clarity + (1|transect)**	**182.0**	**Clarity**	**<0.001**	**0.31**	**0.41**
~(1|transect)	201.2				0.04

*Esox lucius*: t1 (transect 1): 0, 1, 0, 0, 0, 0, 0; t2: 0, 1, 0, 0, 0, 0, 0; t3: 0, 0, 0, 0, 0, 0, 0; t4: 0, 1, 0, 0, 1, 0, 0; t5: 1, 1, 0, 1, 0, 3, 0; t6: 2, 3, 5, 1, 0, 0, 0; t7: 1, 2, 2, 4, 0, 1, 0; t8: 3, 3, 1, 4, 1, 2, 0					
Model	AIC	Reduced	*p*‐value	*R* ^2^ marginal	*R* ^2^ conditional
~Clarity + vegetation + wind speed + temperature + (1|transect)	128.1	Clarity	0.727	0.22	0.49
~Vegetation + wind speed + temperature + (1|transect)	126.2	Vegetation	0.292	0.22	0.50
**~Wind speed + temperature + (1|transect)**	**125.4**	**Wind speed**	**0.040**	**0.16**	**0.49**
~Temperature + (1|transect)	128.0	Temperature	0.003	0.12	0.43
~(1|Transect)	136.8				0.26

*Note*: The best explaining model based on AIC is highlighted in bold. Eight transects were measured seven times each (*N* = 56). Reduced = environmental variable with the highest *p* value reduced from the model. Marginal R^2^ = the variance explained by fixed effects. Conditional *R* ^2^ = the variance explained by the entire model. The number of fish observed in each transect, listed in chronological order of counting trials, is provided after each species name.

The repeatability for the observed density of *A. brama* was weak but statistically significant (ICC = 0.19, 95% confidence interval [CI] = 0.03–0.57, *p* = 0.006). Rejection of two poor vision occasions slightly improved repeatability (ICC = 0.25, 95% CI = 0.02–0.66, *p* = 0.016). ICC for the observed density of *E. lucius* was also weak (ICC = 0.31, 95% CI = 0.09–0.70, *p* < 0.001) with the removal of the two poor vision occasions increasing it (ICC = 0.44, 95% CI = 0.15–0.80, *p* < 0.001).

Visual night‐time counting provides a promising method for assessing the presence and density of large individuals in species such as *A. brama* and *E. lucius* in clear‐water lakes. Both species appeared stationary in the littoral zone in darkness, which made individual counting possible. Water temperature had no effect on the observed abundance of *A. brama* in transects, with temperature ranging from 17.3°C in early September to 2.4°C in late October, but the method is limited to autumn period due to the requirement of darkness. Observed abundance of *E. lucius* in transects was positively related to the water temperature and negatively to the wind speed, suggesting that a rapidly cooling littoral area may be an unpreferred habitat for freshwater *E. lucius* in late autumn.

Compared to the standard gillnet survey results, visual counting was clearly more effective in detecting large *A. brama* individuals and *E. lucius* in general. For example, *E. lucius* was not observed in gillnet surveys at all in 2015 (Paajanen & Kiiskinen, 2015) and was also absent in surveys in the vicinity of the counting sites in 2024 (Huuskonen unpublished). This suggests that night‐time counting can provide supplementary information, especially on the presence of lentic *E. lucius* and large *A. brama*. In addition, we observed species such as bleak *Alburnus alburnus* L., *P. fluviatilis*, roach *Rutilus rutilus* L., and pikeperch *Sander lucioperca* L. during the counting sessions. Hence, the method could be applied to other fishes as well, given species identification can be assured, especially for smaller individuals.

The pilot dataset collected in this study suggests that the presence of *A. brama* in the lake littoral zone has no relationship with the studied environmental variables. *E. lucius* densities were positively related to water temperature and negatively to wind speed. Our results are in line with studies suggesting that *E. lucius* is less present in vegetated littoral zone in winter compared to summer (Jepsen et al., 2001; Rogers, 1998). Wind speed had a negative effect on *E. lucius* observed abundance in the littoral zone, which supports observations by Chapman and Mackay (1984).

*A. brama* abundance estimation had a low repeatability. This may be caused by *A. brama*'s tendency to express sporadic displacements (Kotakorpi et al., 2024; Schulz & Berg, 1987) and as such affect the number of fish that are observable at a given night. *E. lucius* densities were poorly repeatable (Koo & Li, 2016) even when the two poor visibility occasions were removed. In our study, however, fish were not recognized individually and therefore we cannot separate pure density effects from the repeatability of individual behaviors. Visual counting by diving has been used successfully to derive *E. lucius* density estimates (Turner & Mackay, 1985).

Overall, the limiting environmental factors for the use of night‐time counting are wind speed, darkness, and water transparency. Wind conditions are required to be calm to minimize the effect of waves on vision and for certain species preference for shoreline habitat (Chapman & Mackay, 1984). Darkness is required for the use of artificial light, while it may also promote immobility in some species. In high‐latitude regions, required open‐water darkness is achieved in autumn, restricting the use of the method to at most a 4‐month period (from August until ice formation). We recommend that the water transparency metrics (Secchi depth) should exceed the depth of the counted areas because it is essential to be able to distinguish the bottom. This limits the suitability of the method in very humic or otherwise turbid waters. Since we lacked absolute abundance estimates to be compared with the observed densities, future method developments should examine the performance of visual counting against other stock assessment methods. To further generalize the observed counts per studied surface area, the method should be applied in a large set of lakes with repeated assessments. We also pinpoint the potential utility of the method in assessing individual spatial behaviors such as home range sizes in combination with individual external tagging of fish.

## AUTHOR CONTRIBUTIONS

A.T. conceptualized the study and wrote the first draft. A.T. and N.L. participated in field work. A.T., N.L., H.H., and A.V. reviewed and edited the manuscript.
